# An Improved Targeted cAMP Sensor to Study the Regulation of Adenylyl Cyclase 8 by Ca^2+^ Entry through Voltage-Gated Channels

**DOI:** 10.1371/journal.pone.0075942

**Published:** 2013-09-23

**Authors:** Katy L. Everett, Dermot M. F. Cooper

**Affiliations:** Department of Pharmacology, University of Cambridge, Cambridge, United Kingdom; University of Texas Health Science Center at Houston, United States of America

## Abstract

Here we describe an improved sensor with reduced pH sensitivity tethered to adenylyl cyclase (AC) 8. The sensor was used to study cAMP dynamics in the AC8 microdomain of MIN6 cells, a pancreatic β-cell line. In these cells, AC8 was activated by Ca^2+^ entry through L-type voltage-gated channels following depolarisation. This activation could be reconstituted in HEK293 cells co-expressing AC8 and either the α1C or α1D subunit of L-type voltage-gated Ca^2+^ channels. The development of this improved sensor opens the door to the study of cAMP microdomains in excitable cells that have previously been challenging due to the sensitivity of fluorescent proteins to pH changes.

## Introduction

The ubiquitous second messenger cAMP plays critical roles in excitable cells such as neurons, where it has been implicated in processes including long term potentiation [1], pancreatic islet cells, where it plays a role in secretion [2] and cardiomyocytes, where it modulates contractility [3]. Recent studies suggest that the numerous cellular roles of cAMP are orchestrated by organised ‘microdomains’ of cAMP, which may be brought about by scaffolding proteins, cellular organisation or compartmentalisation of the sources of cAMP, the adenylyl cyclases (ACs). The study of these microdomains has been made possible by the development of targetable fluorescent sensors for cAMP. For instance, cAMP at the membrane has been measured using an Exchange protein activated by cAMP (Epac)-based sensor tagged with the lyn kinase palmitoylation sequence [4] and protein kinase A (PKA) fragments targeted using a farnesyltransferase recognition motif [5]. Fluorescent sensors for cAMP have also been targeted to the nucleus using a nuclear localisation sequence and to the mitochondria using the N-terminal sequence of DAKAP1a [6]. However, studies of the regions surrounding individual AC isoforms remain a challenge. These are particularly significant as ACs scaffold some of their downstream targets directly [7,8,9] and conventional global cAMP sensors have been shown to be incapable of measuring cAMP signals in these regions [4].

cAMP is implicated in the regulation of voltage-gated Ca^2+^ channels (VGCCs) and numerous indirect measurements place ACs in intimate association with VGCCs [10,11]. A problem in addressing these milieux, particularly in excitable cells, is that changes in the activity of VGCCs can be accompanied by compensatory transient transitions in pH. Significant decreases in intracellular pH occur following depolarisation of neurons, and pH changes of between 0.3 and 0.5 units have been measured [12,13]. The available fluorescent cAMP sensors are sensitive to small changes in pH [14] rendering studies of AC regulation in excitable cells problematic, since it is the Ca^2+^ influx accompanying depolarisation that is expected to regulate AC activity [15].

Existing FRET based cAMP sensors containing the fluorophores ECFP and EYFP are sensitive to changes in pH, largely due to EYFP which has a pK_a_ of 6.7 [16]. The fluorescence of YFP decreases with decreasing pH, producing an artefact that can be read as an increase in cAMP when using fluorescence ratio-based sensors. Improved yellow fluorophores such as CitrineFP [17] and VenusFP [18] have been developed which are brighter and less pH-sensitive. These have been successfully used as acceptor fluorophores in FRET based cAMP sensors [7,19]. CeruleanFP [20] and mTurquoiseFP [21] were developed as improved cyan fluorescent proteins and are brighter and more stable than CFP, making them potentially better FRET donors in cAMP sensors [22,23].

The Ca^2+^/calmodulin dependent AC8 is a major AC in most brain areas [24]. In non-excitable cells it is regulated by Ca^2+^ entry through store-operated channels (but not by Ca^2+^ released from stores [25]), whereas in excitable cells it is activated by Ca^2+^ entry through VGCCs [26]. The regulation of AC8 by store-operated Ca^2+^ entry has been studied in detail and a direct interaction has been identified between AC8 and the store-operated Ca^2+^ entry channel, Orai1 [27]. The regulation of AC8 by voltage-gated calcium entry has barely been addressed. Exogenously expressed AC8 in the anterior pituitary-derived tumour line, GH _4_C_1_, was activated to a similar extent by store-operated and voltage-gated Ca^2+^ entry, despite the greater global Ca^2+^ concentration that resulted from voltage-gated entry [28]. Given that the brain is a major site of AC8 expression, Ca^2+^ entry through VGCCs is expected to be a major activation mechanism.

cAMP plays an important role in the regulation of insulin secretion from pancreatic β-cells, acting via both PKA and Epac [29,30,31]. β-cells express Ca^2+^-stimulated and Ca^2+^-inhibited ACs, and the expression of AC8 has been identified at the mRNA level in rodent and human cells [32]. Oscillations in cAMP follow stimulation with high glucose, while incretins, such as glucagon-like peptide (GLP)-1, also stimulate cAMP production by binding to G_s_α coupled G-protein coupled receptors [5,33]. Delmeire et al. demonstrated that AC activity in pancreatic β-cells can be synergistically activated by GLP-1 and glucose and that the L-type Ca^2+^ channel blocker verapamil reduced cAMP production in these conditions [34]. As AC8 is capable of being stimulated by both Ca^2+^ and G_s_α it was proposed to be the site at which glucose and incretin signals are integrated. Consequently, the role of AC8 in pancreatic β-cells makes AC8 microdomains a potentially insightful region in which to explore the interaction between cAMP and Ca^2+^ entry through VGCCs.

We developed an improved (Citrine/Cerulean) cAMP sensor fused to the N-terminus of AC8 to study the regulation of the AC8 microdomain in a pancreatic β-cell line, MIN6. A sensor that is insensitive to cAMP binding was utilised as a control for environmental artefacts. Activation of AC8 mediated by Ca^2+^ entry through VGCCs was observed and this effect was reproduced in HEK293 cells co-expressing AC8 and either the α1C or α1D subunits of L-type Ca^2+^ channels. These findings indicate that AC8 microdomains in excitable cells can now be studied in detail.

## Methods

### Constructs

Citrine/Cerulean (Ci/Ce) Epac2-camps was produced by replacement of CFP in Citrine/CFP Epac2-camps [7] with CeruleanFP. The DNA fragment encoding CeruleanFP was generated using KOD Hot Start polymerase (Merck) from Addgene plasmid #18679 [35]. To produce the AC8 targeted sensor, a KpnI site was removed from the Epac2 component of the sensor and KpnI sites were introduced at each end of the sensor by PCR. The whole sensor was subcloned into a KpnI site in the 5′ untranslated region of rat AC8 in pcDNA3 [24]. Removal of the KpnI site and introduction of the R414E mutation in Epac2 and the D416N mutation in AC8 were carried out using the QuikChange protocol (Stratagene) with Phusion High-Fidelity DNA Polymerase (New England Biolabs). GFP-α1C [36] and GFP-α1D [37] were gifts from Dr Gerald Obermair (Innsbruck Medical University, Austria).

### Cell culture and transfection

HEK293 cells were grown in minimum essential medium with 10% (v/v) foetal bovine serum, 2 mM L-glutamine, 100 units/ml penicillin and 100 µg/ml streptomycin. MIN6 cells [38] (from Dr Anders Tengholm (Uppsala University, Sweden)) were grown in Dulbecco’s modified Eagle’s medium, containing 4500 mg/ml glucose, supplemented with 15% (v/v) foetal bovine serum, 2 mM L-glutamine, 100 units/ml penicillin, 100 µg/ml streptomycin and 50 µM 2-mercaptoethanol. All cells were maintained at 37 °C in a humidified atmosphere containing 5% CO_2._

For imaging, HEK293 cells were plated on 25-mm poly-L-lysine coated coverslips 24 h prior to transfection. Cells were transfected with 1-2 µg cDNA using Lipofectamine 2000. For Western blotting, HEK293 cells were plated on 60-mm dishes and transfected with 2.5 µg cDNA. For *in vitro* FRET measurements, cells were plated on 92-mm (targeted sensor) or 150-mm (global sensor) dishes and transfected with 3.5 µg or 10 µg cDNA respectively. Experiments were carried out 48 h post-transfection. For transfection of MIN6 cells, 2x10^5^ cells and 0.2 µg cDNA per coverslip were mixed in solution with Lipofectamine 2000 and then added to 25-mm poly-L-lysine coated coverslips. Experiments were carried out 24 or 48 h post-transfection.

### In vitro FRET measurements

For global sensors, HEK293 cells transiently expressing Epac2-camps were pelleted and lysed in 5 mM Tris, pH 7.3 and 2 mM EDTA by passing though a 21-gauge needle 20 times. Lysates were centrifuged at 20000*g*, 4 °C for 1 h. For calibration of Ci/Ce Epac2-camps AC8, crude membranes were prepared from transfected cells. The cells were pelleted and resuspended in homogenisation buffer [2 mM MgCl_2_, 1 mM EDTA, 1 mM phenylmethylsulphonyl fluoride, 1 mM benzamidine, 1 µg DNase, and 50 mM Tris, pH 7.4] by passing through a 21-gauge needle 20 times. Following centrifugation at 200*g*, the supernatant was transferred to a new tube and centrifuged at 15000*g* for 15 min. The membrane-containing pellet was resuspended in 40 mM Tris, pH 7.3. Subsequently the fluorescence emission spectra of the lysates and membranes were measured (excitation at 436±8 nm, emission from 450 to 550 nm) in a LS50B spectrofluorimeter (PerkinElmer) and increasing concentrations of cAMP were added. Concentrations of cAMP were spectrofluorometrically established at λ_259 nm_ and sigmoidal dose-response curves were obtained using GraphPad Prism version 4 (GraphPad Software, Inc).

### Confocal microscopy

Transfected HEK293 or MIN6 cells were imaged using a Leica SP5 TCS laser scanning confocal microscope attached to a DM16000 inverted microscope (63x oil immersion objective). Epac2-camps sensors were excited at 514 nm and emission was collected between 520 and 560 nm.

### RT-PCR

Total RNA was prepared from MIN6 cells using TRI Reagent (Sigma) according to the manufacturer’s instructions. 1 µg RNA was transcribed to cDNA using SuperScript II Reverse Transcriptase (Invitrogen). DNA fragments were amplified from MIN6 cDNA using Taq DNA polymerase (New England Biolabs). Primer sequences were AC1, 5′ CCTTTTGGTCACCTTCGTGT and 3′ CATCTCCACACAGCAGTGG, AC6, 5′ GCATCCTAGCAGCCGTGC and 3′ CAGACATCAAACTGCCATTTC, and AC8, 5′ GCCAGAGGCGCAAATCGG and 3′ GGTAAATCCTTTGACATCTGC. Reactions containing 1 ng of plasmid DNA encoding the respective full length cDNAs were carried out as positive controls. Amplified fragments were separated on a 1.2% agarose gel and visualised with GelRed nucleic acid gel stain (Biotium). Digital images were generated with the GeneFlash gel documentation system (Syngene).

### Single cell Ca^2+^ measurements

HEK293 or MIN6 cells were loaded with 2 µM fura-2 AM and 0.01% Pluronic F-127 (Life Technologies) for 35 min at room temperature. For HEK293 experiments, extracellular buffered saline contained 140 mM NaCl, 4 mM KCl, 1 mM CaCl_2_, 0.2 mM MgCl_2_, 11 mM D-glucose and 10 mM HEPES, pH 7.4. For MIN6 experiments, extracellular buffered saline contained 125 mM NaCl, 4.8 mM KCl, 1.3 mM CaCl_2_, 1.2 mM MgCl_2_, 3 mM D-glucose and 25 mM HEPES, pH 7.4. After loading, cells were washed several times and then imaged using a CoolSNAP-HQ CCD camera (Photometrics) and monochromator system (Cairn Research) attached to a Nikon Eclipse Ti microscope (40x oil immersion objective). Emission at 535 nm was measured following 340 and 380 nm excitation. Images were collected at 1 Hz using MetaFluor software (Molecular Devices). For buffers containing high KCl, monovalent cation concentration was maintained at 144 or 129.5 mM but with variable amounts of KCl and NaCl as indicated. Single cell Ca^2+^ measurements were plotted as changes in the 340/380 nm excitation ratio relative to the maximum change seen upon addition of 4 µM ionomycin and 10 mM CaCl_2._

### Single cell pH measurements

pH measurements were performed using the same system as for single cell Ca^2+^ measurements. HEK293 cells were loaded with 2 µM BCECF (Life Technologies) for 35 min at room temperature and then washed several times before imaging. Emission at 535 nm was measured following excitation at 440 and 490 nm and images were collected at 1 Hz using MetaFluor software. For calibration, minimum and maximum values were obtained by addition of HCl and NaOH following treatment with 5 µM FCCP. pH values were calculated using the formula pH = pK_a_-log[(R_max_-R)/(R–R_min_)].

### Epac2-camps FRET measurements

HEK293 or MIN6 cells expressing the global or AC8-targeted sensors were imaged in extracellular buffered saline as described for Ca^2+^-imaging experiments using an Andor Ixon+ EMCCD camera and an Optosplit (505DC) to separate CFP/CeruleanFP (470 nm) and YFP/CitrineFP (535 nm) emission images (Cairn Research). Cells were excited at 435 nm using a monochromator (Cairn Research) and 51017 filter set (Chroma Technology Corp.) attached to a Nikon Eclipse TE2000-S microscope (40x oil immersion objective). Emission images were collected every 3 s and analysed using MetaMorph imaging software (Molecular Devices). Cells in which the YFP/CitrineFP fluorescence intensity was less than twice the background signal were excluded, as were cells with excessive expression of the fluorescent probe. Single cell FRET data were plotted as changes in background subtracted 470 nm *versus* 535 nm (CFP/YFP or Cerulean/Citrine) emission ratio relative to the initial ratio or to the maximum FRET ratio change seen with saturating cAMP concentrations (achieved with 10 µM forskolin, 100 µM 3-isobutyl-1-methylxanthine and 10 µM isoproterenol (for HEK293) or 100 nM prostaglandin E1 (for MIN6)).

### Preparation of HEK293 lysate and Western blotting

Transfected cells in 60-mm dishes were washed once in cold phosphate buffered saline and lysed in 250 µl lysis buffer [10% (v/v) glycerol, 100 mM NaCl, 50 mM Tris, pH 7.4, 0.3% NP40, 100 µM EGTA, 2 mM dithiothreitol, 1 mM phenylmethylsulphonyl fluoride, 1 mM benzamidine, 1x protease inhibitor cocktail (Sigma), 10 mM β-glycerophosphate and 2 mM sodium orthovanadate] by rotating for 30 min at 4 °C and then passing through a 21-gauge needle 10 times. The lysates were centrifuged at 400*g* for 5 min. Laemmli buffer was added to the supernatant and the samples were incubated at 37 °C for 30 min prior to Western blotting.

Proteins were resolved on an 8% SDS-polyacrylamide gel and then transferred to nitrocellulose membrane. Anti-GFP or anti-α-tubulin antibodies (Sigma) were diluted 1:5000 and goat anti-mouse IgG conjugated to horseradish peroxidase (Promega) was diluted 1:20000. The membrane was visualised with ECL Prime (GE Healthcare) using a BioSpectrum imaging system (UVP).

## Results

**Figure 1:**
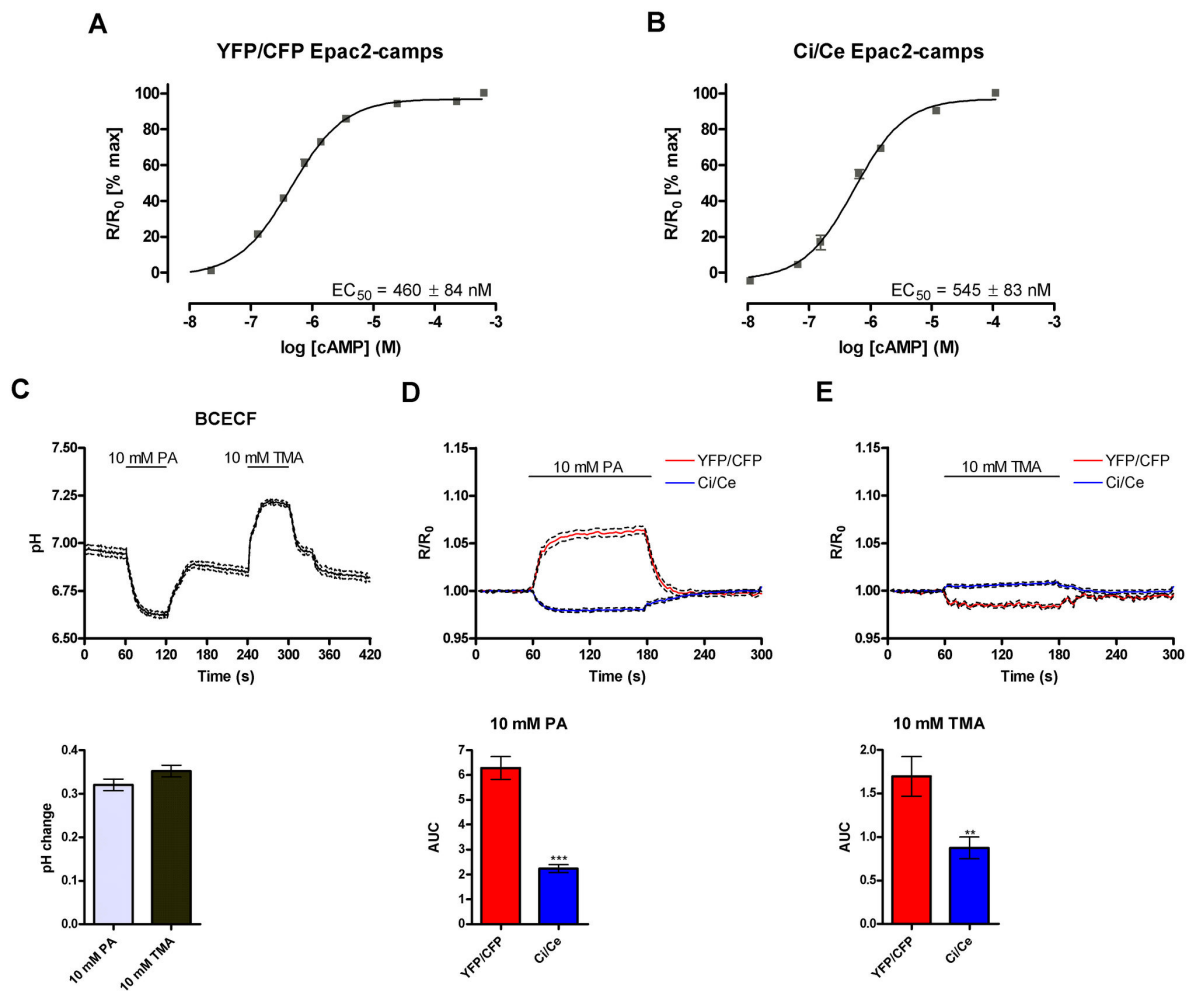
Development of a cAMP sensor with decreased pH sensitivity. (**A**) and (**B**) The sensitivity of the global cAMP sensors was determined *in* *vitro* by addition of increasing concentrations of cAMP to lysates prepared from HEK293 cells expressing the sensor. EC_50_ presented as mean±SD (n=3 or 4). (**C**) BCECF was used to measure the pH change produced by addition of 10 mM propanoic acid (PA) or 10 mM trimethylamine (TMA). The pH change is quantified in the lower panel (mean±SEM; n=107 cells from two independent experiments). (**D**) and (**E**) Normalised fluorescent ratios from HEK293 cells expressing ‘dead’ global sensors treated with 10 mM PA or 10 mM TMA. The area under the curve (AUC) is shown in the lower panel (mean±SEM; n=33 (YFP/CFP (PA)), 63 (Ci/Ce (PA)), 25 (YFP/CFP (TMA)) and 43 (Ci/Ce (TMA)) cells. Data from 4-6 independent experiments). ** p<0.001, *** p<0.0001 compared to YFP/CFP measured by t test.

To improve the Epac2-camps cAMP sensor [39], EYFP and ECFP were replaced with the fluorophores, CitrineFP and CeruleanFP, to produce Ci/Ce Epac2-camps, which was expected to be less sensitive to small changes in pH and chloride [22]. *In vitro* calibration of the YFP/CFP and Ci/Ce sensors using lysate from transfected HEK293 cells showed that their EC_50_ values for cAMP were similar (460±84 nM and 545±83 nM respectively (mean±SD, n=3 or 4); Figure 1A and B).

Treatment of HEK293 cells with weak acid or base alters the internal pH [40]. The ratiometric pH dye BCECF was used to demonstrated that treatment of HEK293 cells with 10 mM propanoic acid (PA) or 10 mM trimethylamine (TMA) produced pH changes of −0.32±0.01 and 0.35±0.01 respectively (mean±SEM, n=107; Figure 1C). Introduction of an inactivating mutation, R414E (numbering from full length Epac2), into the cAMP binding domain of the sensor prevents cAMP binding [41]. The ‘dead’ versions of the YFP/CFP and Ci/Ce sensors were expressed in HEK293 cells, which were then treated with 10 mM PA or 10 mM TMA to alter internal pH (Figure 1D and E). As the ‘dead’ sensor is insensitive to cAMP any change in the fluorescence ratio can be attributed to environmental artefacts; in this case, changes in pH. In cells transfected with Ci/Ce Epac2-camps a significantly smaller change in the fluorescence ratio accompanied the pH decrease or increase, compared to the YFP/CFP pair. The direction of the fluorescence ratio change is also altered when the fluorophores are switched. These data confirmed that we had produced a less pH sensitive sensor.

**Figure 2:**
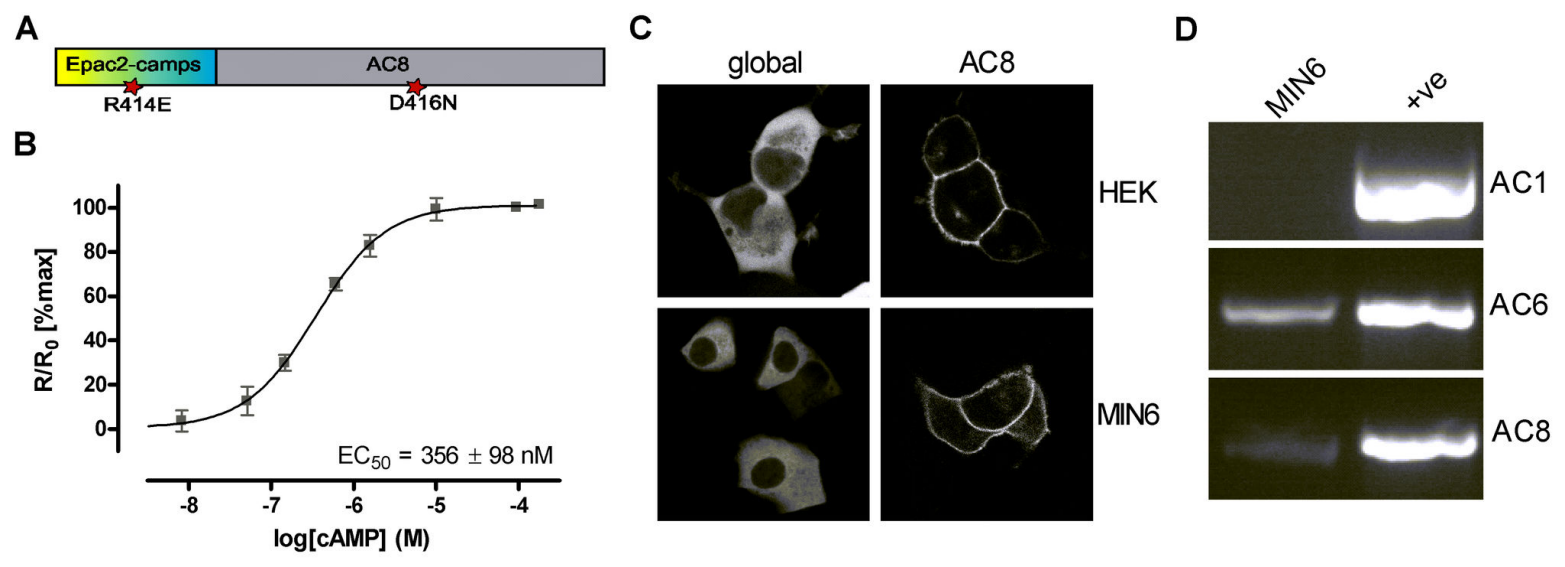
Development of an AC8-targeted sensor for use in MIN6. (**A**) Cartoon showing the Citrine/Cerulean sensor fused to the N-terminus of AC8 with inactivating mutations in Epac2-camps and AC8 indicated. (**B**) The cAMP sensitivity of the targeted sensor was determined *in* *vitro* by addition of increasing concentrations of cAMP to crude membranes prepared from HEK293 cells expressing the sensor. EC_50_ presented as mean±SD (n=4). (**C**) Confocal images of HEK293 and MIN6 cells expressing the global and AC8 targeted forms of the Ci/Ce sensor. (**D**) RT-PCR to identify Ca^2+^-regulated adenylyl cyclases in MIN6 cDNA and in positive control reactions.

Ci/Ce Epac2-camps was fused to the N-terminus of AC8 to permit study of the AC8 microdomain (Figure 2A). *In vitro* calibration of the targeted sensor using membranes from transfected HEK293 cells showed that its EC_50_ for cAMP was not greatly altered from the cytosolic global sensor (545±83 nM – global, compared to 356±98 nM – AC8-targeted (mean±SD, n=3 or 4); Figure 2B). In contrast, a previously described AC8-targeted form of the YFP/CFP sensor displayed reduced cAMP sensitivity [4]. When the global and AC8-targeted Ci/Ce sensors were expressed in HEK293 cells the global sensor was expressed throughout the cytosol but excluded from the nucleus, whereas the targeted sensor was localised to the membrane (Figure 2C, top panels).

Further studies using the new sensor were carried out in the MIN 6 cell line, a murine pancreatic β-cell surrogate that has been reported to express AC8 [7,42]. RT-PCR confirmed that the cells express AC8 in addition to the Ca^2+^-inhibited AC6, but not the Ca^2+^-stimulated AC1 (Figure 2D). When the global and targeted sensors were expressed in MIN6 cells they showed comparable localisation to that seen in HEK293 cells (Figure 2C, bottom panels).

**Figure 3:**
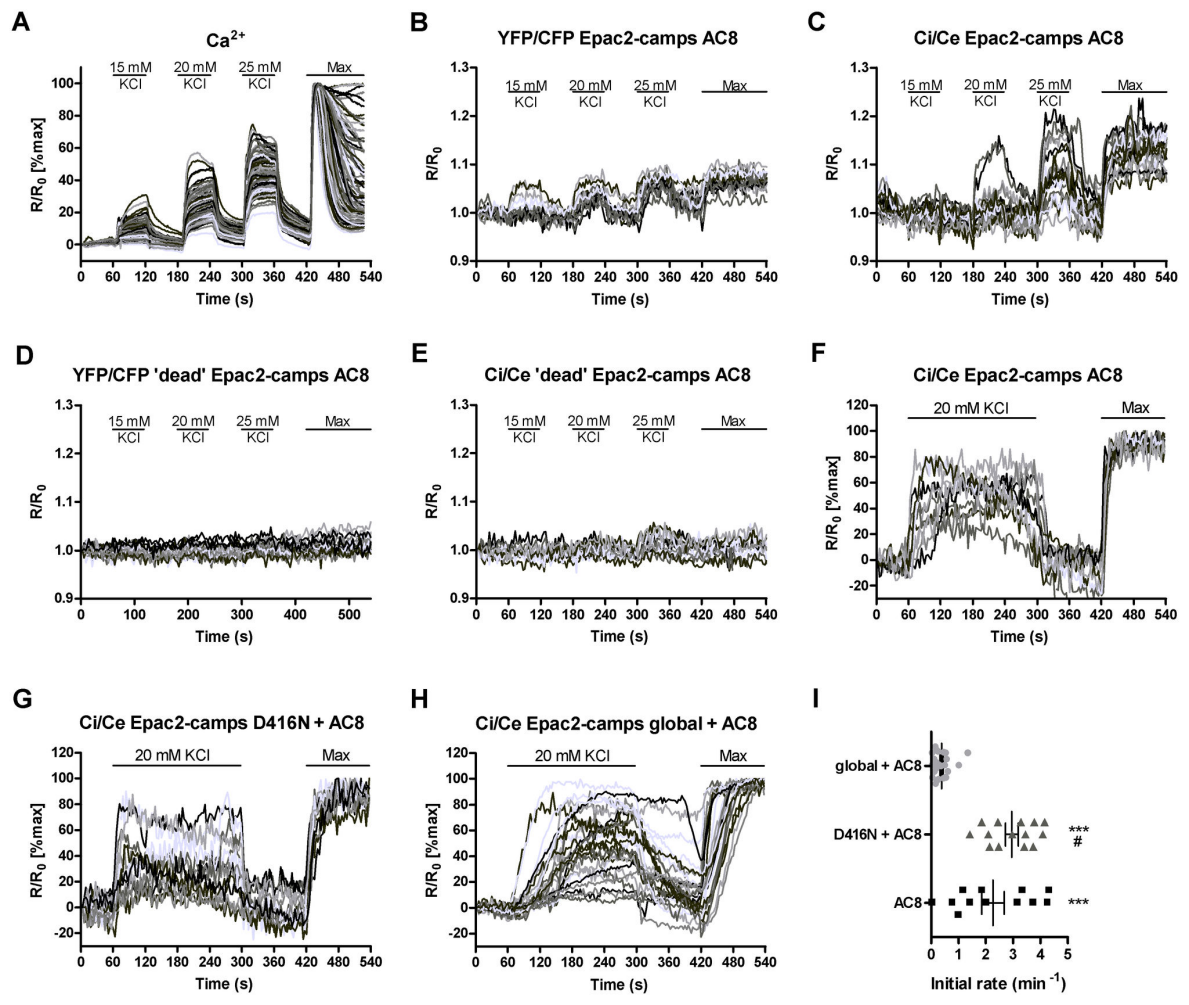
Characterisation of the AC8-targeted sensor in MIN6 cells. (**A**) Ca^2+^ measurements in fura-2 loaded MIN6 cells depolarised with increasing concentrations of KCl in the presence of 1.3 mM Ca^2+^. Traces represent individual cells (n=96 cells from a representative experiment) (**B**)-(**E**) cAMP measurements in MIN6 cells expressing the indicated sensor and depolarised with increasing concentrations of KCl in the presence of 1.3 mM Ca^2+^. Traces represent individual cells ((**B**) n=15 cells, (**C**) n=19 cells, (**D**) n=19 cells and (**E**) n=11 cells from 5-6 independent experiments). (**F**) -(H) cAMP measurements in MIN6 cells expressing the indicated constructs and depolarised with 20 mM KCl in the presence of 1.3 mM Ca^2+^. Traces represent individual cells ((**F**) n=11 cells, (**G**) n=13 cells and (**H**) n=26 cells, from 5-7 independent experiments). (**I**) The initial rate of FRET change for each cell represented in panels (**F**)-(**H**) was calculated and shown a scatter plot with the mean and SEM. Data was normalised between the time point before depolarisation and the peak reached within the first two minutes following depolarisation. The initial rate of FRET change was calculated over the first 12 s (five data points) for each cell. Cells that did not respond to depolarisation were excluded from this analysis. *** p<0.0001 compared to global+AC8, # p>0.05 compared to AC8 measured by ANOVA with Tukey’s post test.

Depolarisation of MIN6 cells with 15-25 mM KCl leads to Ca^2+^ entry, as measured by fura-2 (Figure 3A). The increase in global Ca^2+^ concentration is reversible and depends on the KCl concentration. This system was used to further characterise the AC8-targeted cAMP sensor and compare it to the previously developed YFP/CFP Epac2-camps AC8 [4]. In experiments using the targeted sensor care was taken to select cells for analysis in which the sensor had been expressed at the membrane. Depolarisation increasing concentrations of KCl resulted in increasing cAMP production (Figure 3B and C). The production of cAMP occurred rapidly following depolarisation and cAMP levels decreased rapidly following repolarisation. The new Ci/Ce sensor has a larger range (ΔR/R_0_) than the targeted YFP/CFP sensor when maximum stimulation is applied (average ΔR/R_0_ is 0.134±0.008 compared to 0.069±0.005 (mean±SEM, n=22 or 15 cells respectively)), yielding an improved signal to noise ratio. Cell variability was higher with the new sensor but this is likely due to greater variability in expression levels. In order to verify that the response following depolarisation reflected cAMP binding rather than a pH artefact, the same experiment was carried out using cells transfected with the targeted ‘dead’ sensors. There was almost no response when using these sensors, which confirmed that these effects were not due to environmental artefacts (Figure 3D and E). The lack of artefact seen with the YFP/CFP ‘dead’ sensor in Figure 3D indicates that MIN6 cells do not undergo significant pH shifts in the AC8 microdomain following depolarisation. This observation suggests that the improvements in the performance of the sensor in this system are likely due to effects such as improved expression and/or improved optical properties of the fluorophores.

The AC8-targeted sensor used here contains full-length active AC8 in the same molecule as the cAMP sensor. To confirm that the cAMP response can be replicated in a situation where the enzyme and sensor are not physically linked, a sensor containing an inactivating mutation in the catalytic domain of AC8 (AC8 D416N) [4] was co-expressed with wild-type AC8 (Figure 3G). Analysis of the initial rates of FRET change (Figure 3I) showed no significant difference between the two conditions, demonstrating that the response following depolarisation did not depend on the fusion of the sensor and active enzyme. Measurements using the global sensor and overexpressed AC8 show a slower cAMP response than with the targeted sensors. This suggests that the cAMP produced by AC8 may need to diffuse away from its site of production before it can be detected by the global sensor (Figure 3H and I). To confirm that the difference in the rate of FRET change was not due to differences in sensor kinetics, control experiments were performed *in vitro* (Figure S1). Addition of 250 nM or 1 µM cAMP to either sensor resulted in a rapid decrease in YFP fluorescence and there was no difference in the speed of response between the two sensors. The different responses measured with the targeted and global sensors in MIN6 cells therefore support the existence of an AC8 microdomain in which changes in cAMP levels occur more rapidly than those seen in the whole cell.

**Figure 4:**
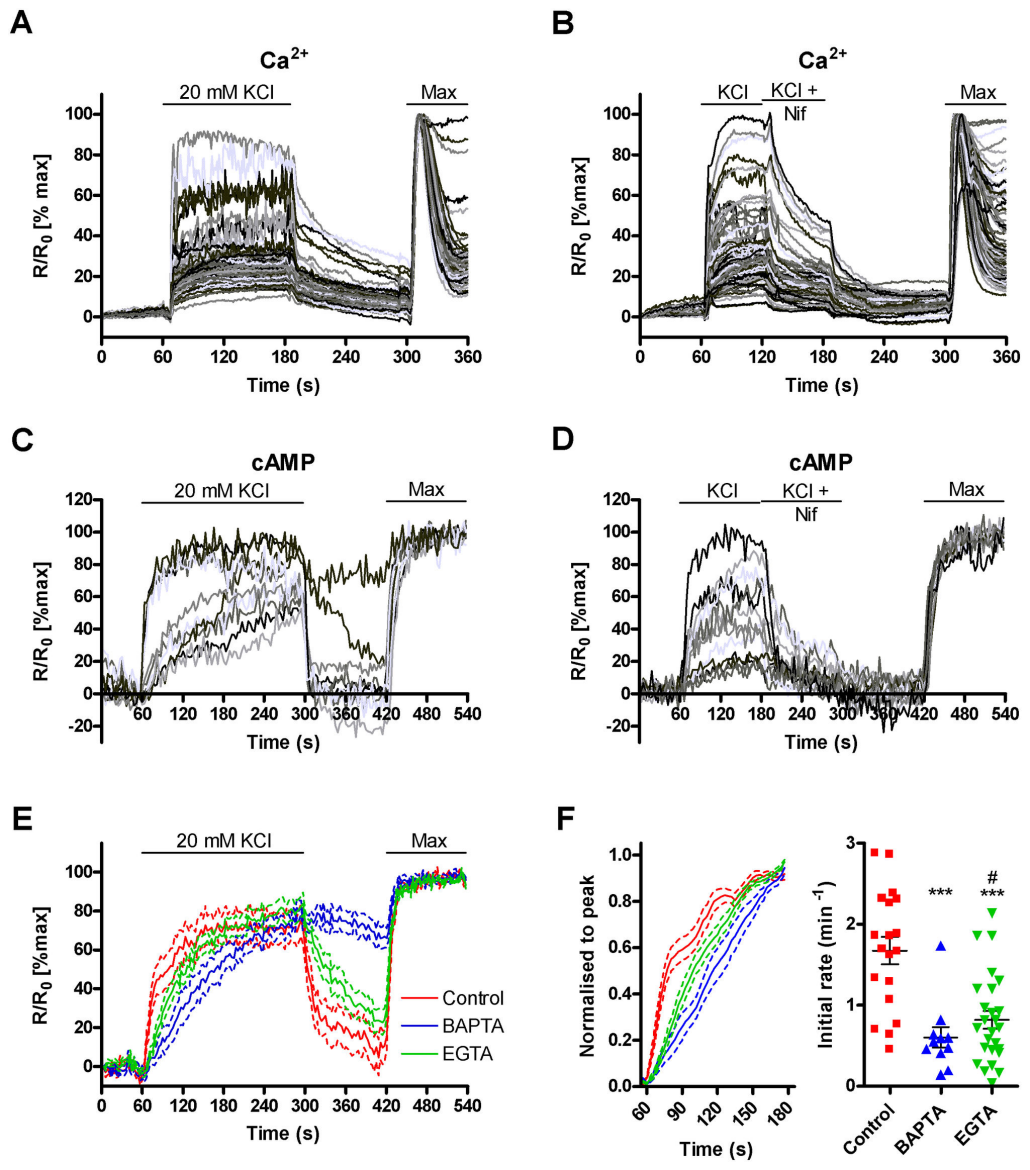
Ca^2+^ entry through VGCCs in MIN6 activates AC8. (**A**) Ca^2+^ measurements in fura-2 loaded MIN6 cells depolarised with 20 mM KCl in the presence of 1.3 mM Ca^2+^. Traces represent individual cells (n=102 cells from a representative experiment) (**B**) Ca^2+^ entry through L-type VGCCs was blocked with 10 µM nifedipine. Traces represent individual cells (n=102 cells from two representative experiments) (**C**) cAMP measurements in MIN6 cells expressing Ci/Ce Epac2-camps AC8 and depolarised with 20 mM KCl in the presence of 1.3 mM Ca^2+^. Traces represent individual cells (n=10 cells from 3 independent experiments) (**D**) Ca^2+^ entry through L-type VGCCs was blocked with 10 µM nifedipine during cAMP measurements. Traces represent individual cells (n=14 cells from 3 independent experiments) (**E**) Average cAMP response from MIN6 cells expressing Ci/Ce Epac2-camps AC8 and pre-loaded for 30 min with 30 µM BAPTA-AM or EGTA-AM (control contains no chelator) (dotted lines represent SEM). n=20 (control), 11 (BAPTA-AM) and 26 (EGTA-AM) cells from 8-10 independent experiments. (**F**) Data from (**E**) was normalised between the time point before depolarisation and the peak reached within the first two minutes following depolarisation. The initial rate of FRET change was calculated over the first 12 s (five data points) for each cell and shown on a scatter plot with the mean and SEM. Cells that did not reach 25% of the maximum response were excluded from this analysis. *** p<0.0001 compared to control, # p>0.05 compared to BAPTA-AM measured by ANOVA with Tukey’s post test.

The Ca^2+^ entry that occurs following depolarisation is likely to be due to the activity of L-type channels, as these are the major VGCCs expressed in murine pancreatic β-cells [43]. To test this hypothesis the effectiveness of an L-type channel antagonist, nifedipine, was assessed. The depolarisation induced Ca^2+^ entry into MIN6 cells is greatly reduced by addition of 10 µM nifedipine, confirming that the majority of Ca^2+^ entry is through these channels (Figure 4A and B). Production of cAMP following depolarisation is also greatly reduced by nifedipine (Figure 4C and D) demonstrating that cAMP production by AC8 results from Ca^2+^ entry through L-type VGCCs. The effects of nifedipine on Ca^2+^ entry appear slower that those on cAMP production. This difference is likely due to the difference between global Ca^2+^ measurements and localised cAMP measurements. Fura-2 and nifedipine both absorb light at 340 nm and nifedipine absorption has been linked to the production of cytotoxic cleavage products [44]. To control for artefactual effects of nifedipine absorption on fura-2 based Ca^2+^ measurements, HEK293 cells with and without fura-2 were imaged in the presence and absence of 10 µM nifedipine (Figure S2). No changes in the 340/380 nm ratio were seen upon addition or removal of nifedipine. As the concentrations of nifedipine used were fairly low and the experiments were short there were unlikely to be cytotoxic effects of any cleavage products.

The relative effectiveness of the Ca^2+^-chelators BAPTA and EGTA can be used to estimate the functional range between a Ca^2+^ source and a Ca^2+^ sensitive molecule [45]. The two chelators have similar affinities for Ca^2+^ but the on-rate for BAPTA is ~150 times faster than that for EGTA. This means that EGTA is less effective at preventing Ca^2+^ diffusion from its site of entry than BAPTA. Comparisons of the effects of BAPTA and EGTA have previously been used to demonstrate that both AC6 and AC8 are localised close to the site of store-operated Ca^2+^ entry [46,47]. In MIN6 cells, pre-incubation with membrane permeant forms of the chelators, BAPTA-AM and EGTA-AM, led to a decreased initial rate of cAMP production following depolarisation compared to control (Figure 4E and F and Figure S3). However, there was no statistically significant difference between the effects of BAPTA and EGTA in attenuating the initial rate of cAMP production. This may suggest that AC8 and the sites of voltage-gated Ca^2+^ entry are not as closely associated as between AC8 and the sites of store-operated Ca^2+^ entry, although the magnitude of the Ca^2+^ signal entering through voltage-gated Ca^2+^ channels may be far greater than through store-operated Ca^2+^ channels where BAPTA has been shown to be more efficacious than EGTA both at precluding AC6 regulation by store-operated Ca^2+^ entry and the detection of Ca^2+^ signals by a GCamp2-tagged AC8 [46,47].

**Figure 5:**
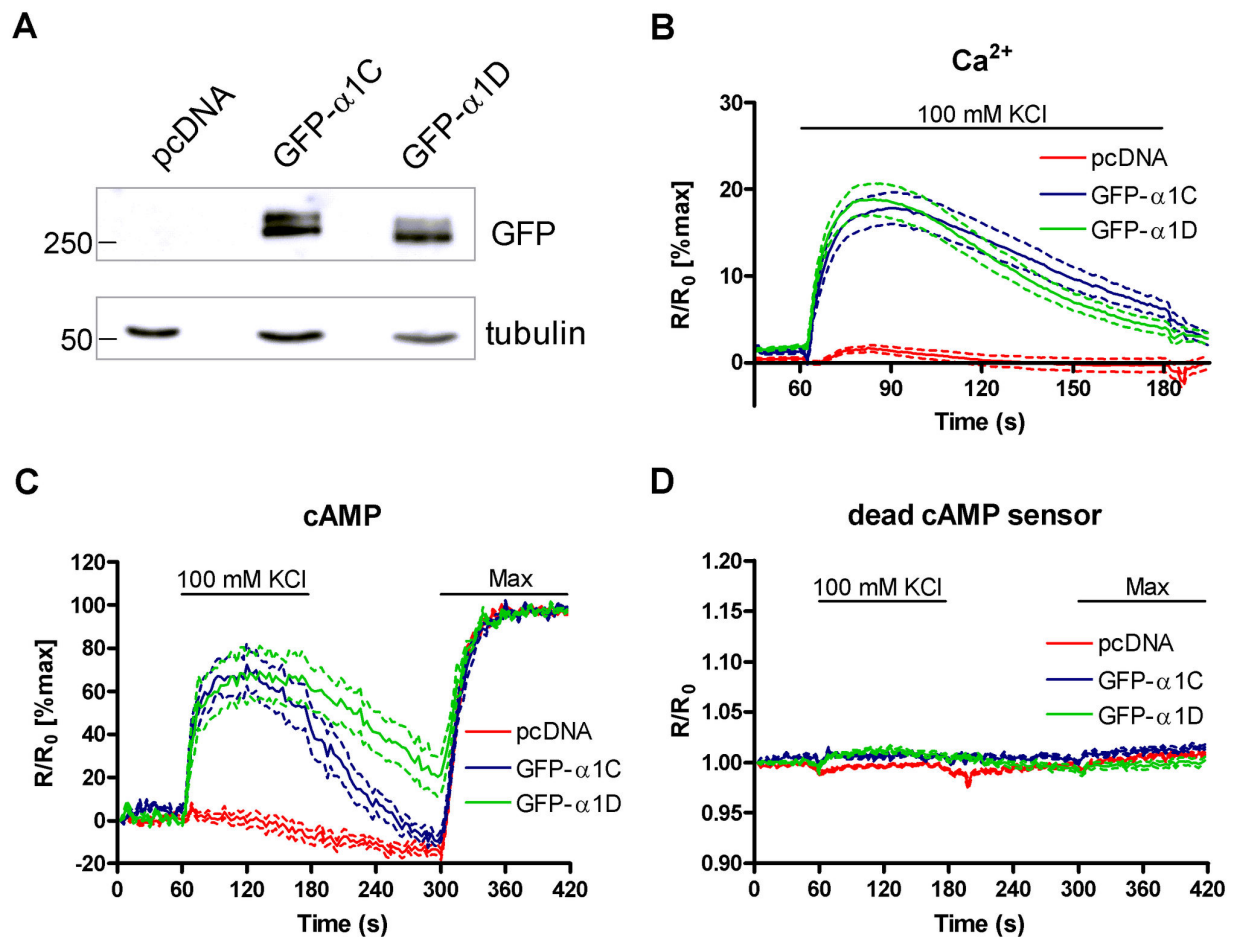
Overexpression of VGCCs in HEK293 facilitates AC8 activation. (**A**) Western blot demonstrating the overexpression of GFP-α1C and GFP-α1D in HEK293 cells. Tubulin was used as a loading control. (**B**) Ca^2+^ measurements in fura-2 loaded HEK293 cells transfected with empty vector, GFP-α1C or GFP-α1D. Cells were depolarised with 100 mM KCl in the presence of 2 mM Ca^2+^. Cells which showed no response were assumed not to express the channel subunit and so were excluded from the analysis. n=150 (pcDNA), 39 (GFP-α1C) and 57 (GFP-α1D) cells from 3-4 independent experiments. (**C**) cAMP measurements in HEK293 cells co-expressing Ci/Ce Epac2-camps AC8 and the indicated channel. Cells were depolarised with 20 mM KCl in the presence of 2 mM Ca^2+^. n=28 (pcDNA), 17 (GFP-α1C) and 18 (GFP-α1D) cells from 4-5 independent experiments. (**D**) Control measurements using cells co-expressing Ci/Ce Epac2-camps ‘dead’ AC8 and the indicated channel. n=24 (pcDNA), 18 (GFP-α1C) and 24 (GFP-α1D) cells from 4 independent experiments. On all graphs dotted lines represent SEM.

Pancreatic β-cells express both Ca_v_1.2 and Ca_v_1.3 channels [43,48], which contain the α1C and α1D subunits respectively. Overexpression of GFP-α1C and GFP-α1D in HEK293 cells produced bands of expected size on a Western blot (Figure 5A). The two channels express at similar levels, with no evidence of degradation. It has previously been shown that expression of the α1C subunit in HEK293 cells facilitates Sr^2+^ entry upon depolarisation with high KCl [49]. Depolarisation of HEK293 cells with 100 mM KCl resulted in Ca^2+^ entry through exogenously expressed α1C and α1D (Figure 5B and Figure S4A-C). Expression of either channel resulted in similar levels of Ca^2+^ entry and the lack of response in cells transfected with empty vector demonstrated that the Ca^2+^ entry depended on channel expression. In response to depolarisation, cAMP increases in HEK293 cells expressing the α1C and α1D subunits, as measured with the AC8-targeted sensor (Figure 5C and Figure S4D-F). In some cells the high levels of cAMP produced saturated the sensor. It is therefore not possible to assess whether there is a difference between the amount of cAMP produced in response to Ca^2+^ entry through α1C and α1D. The use of the targeted sensor and the absence of Ca^2+^ stimulated AC activity in our HEK293 clone [27] indicate that this cAMP production is due to AC8 activity. Experiments carried out using the ‘dead’ targeted sensor confirmed that this response was not due to an environmental artefact (Figure 5D and Figure S4G-I). These data demonstrate that the presence of an L-type α subunit and AC8 is sufficient for voltage-gated Ca^2+^ entry to trigger cAMP production.

## Discussion

We have developed a new AC8-targeted cAMP sensor, Ci/Ce Epac2-camps AC8, which is an improvement on the previous targeted cAMP sensor both because of the more desirable properties of the alternative fluorophores and the increased dynamic range of the sensor. Although the reduced pH sensitivity of the CitrineFP/CeruleanFP fluorophore pair was not important for its use in MIN6 cells, this quality opens up the possibility of using the sensor to study AC8 activity in excitable cells, particularly neurons, where pH changes upon depolarisation might be expected to alter fluorescence in pH-sensitive fluorophores.

A recent report suggested that AC1 is the key Ca^2+^-regulated AC in MIN6 cells [50]; however we saw no evidence of AC1 expression in our MIN 6 cell clone. Other studies have suggested a role for AC8 in pancreatic β-cells, such as its activation by glucose in the rat β-cell line, RINm5F [34]. Although AC8 activity has previously been shown to be activated following store-operated Ca^2+^ entry in MIN6 cells [42] the present study is the first to show that AC8 can be activated by depolarisation of MIN6 cells and that this activation is due to Ca^2+^ entry through L-type VGCCs.

The equivalent efficacy in the attenuation of the rate of cAMP production caused by the two Ca^2+^-chelators, BAPTA and EGTA, suggests that AC8 and the VGCC may not be localised as closely together as are AC8 and Orai1 in non-excitable cells [27]. This would provide one explanation for the previous finding in GH _4_C_1_ cells that store-operated and voltage-gated Ca^2+^ entry led to equivalent levels of cAMP production even though the Ca^2+^ entry through voltage-gated channels was greater [26]. It is conceivable that the amount of Ca^2+^ entering through L-type channels in these experiments is so high that even if there were a direct interaction between some channels and AC8, the chelation of global Ca^2+^ that is achievable by the BAPTA/EGTA-AM loading – perhaps no more than hundreds of micromolar – is incapable of intercepting sufficient Ca^2+^ to block AC8 activation. The lower amount of Ca^2+^ entering through store-operated channels can be intercepted by BAPTA-AM in HEK293 cells expressing exogenous AC8 [47], however when, for instance, Ca^2+^-dependent inactivation of L-type channels is attenuated by BAPTA, this is only achieved in attached cell-patches where extremely high millimolar concentrations of chelator can be applied [51]. Both AC8 and Ca_v_ channels have been identified in lipid rafts [48,52,53] suggesting that they may be localised to the same type of general membrane domains. In addition both AC8 and α1C bind to the scaffolding protein AKAP79/150 [7,54] providing a possible indirect mechanism for the two proteins to interact.

Co-expression of AC8 and α1C or α1D in HEK293 cells resulted in depolarisation dependent activation of AC8. This confirms that Ca^2+^ entry through L-type VGCCs is sufficient for activation and no difference between the efficiency of activation via Ca_v_1.2 or Ca_v_1.3 was identified. α1C and α1D are both phosphorylated by PKA leading to increased Ca^2+^ entry [55,56]. Since AC8 and Ca_v_ channels bind to the PKA scaffold AKAP79/150, an extremely efficient mechanism can be envisaged where synergistic positive feedback is triggered by the cAMP produced by AC8 and the Ca^2+^ entering in response to phosphorylation of the channel by the PKA. The availability of the targeted sensor described here opens the door to dissecting the influences upon this microdomain, particularly in neuronal contexts where the improved pH sensitivity of the sensor will be useful.

## Supporting Information

Figure S1 **Kinetics of the global and AC8-targeted sensors.** The kinetics of the global and AC8-targeted sensors were compared *in* *vitro* using lysate and membranes prepared from HEK293 cells expressing the sensors. YFP emission was measured at 535 nm every 0.1 s following CFP excitation at 435 nm and (**A**) 250 nM or (**B**) 1 µM final concentrations of cAMP were added. Mean traces are shown, n=3 for global and 2 for AC8-targeted sensor.
(TIF)

Figure S2 **Control data for nifedipine imaging.** HEK293 cells (**A**) loaded with fura-2 or (**B**) without fura-2 were imaged at 340 nm and 380 nm and the ratio calculated. 10 µM nifedipine was added for 60 s. n=197 (fura-2) or 91 (no fura-2) cells from three independent experiments.
(TIF)

Figure S3 **Single cell data for cAMP measurements in control, BAPTA-AM and EGTA-AM conditions.** cAMP measurements in MIN6 cells expressing Ci/Ce Epac2-camps AC8 and depolarised with 20 mM KCl in the presence of 1.3 mM Ca^2+^. Traces represent individual cells from 8-10 independent experiments. (**A**) control conditions (n=20 cells) or pre-loaded for 30 min with (**B**) 30 µM BAPTA-AM (n=11 cells) or (**C**) 30 µM EGTA-AM (n=26 cells).
(TIF)

Figure S4 **Single cell data for Ca^2+^ and cAMP measurements in cells expressing VGCCs.** Cells were depolarised with 100 mM KCl in the presence of 2 mM Ca^2+^. (**A**) -**(C)** Ca^2+^ measurements in fura-2 loaded HEK293 cells transfected with empty vector (n=35 cells), GFP-α1C (n=24 cells) or GFP-α1D (n=34 cells). Traces represent individual cells and are representative of 3-4 independent experiments. (**D**) -**(F)** cAMP measurements in MIN6 cells co-expressing Ci/Ce Epac2-camps AC8 and empty vector (n=28 cells), GFP-α1C (n=17 cells) or GFP-α1D (n=18 cells). Traces represent individual cells from 4-5 independent experiments. (**G**) -**(I)** cAMP measurements in MIN6 cells co-expressing Ci/Ce Epac2-camps ‘dead’ AC8 and empty vector (n=24 cells), GFP-α1C (n=18 cells) or GFP-α1D (n=24 cells). Traces represent individual cells from 5 independent experiments.
(TIF)
